# Race, ethnicity, and the use of regional anesthesia in cancer patients undergoing open abdominal surgery: A single-center retrospective cohort study

**DOI:** 10.3389/fmed.2022.950444

**Published:** 2022-08-18

**Authors:** Pascal Owusu-Agyemang, Lei Feng, Vivian H. Porche, Uduak U. Williams, Juan P. Cata

**Affiliations:** ^1^Department of Anesthesiology and Perioperative Medicine, The University of Texas MD Anderson Cancer Center, Houston, TX, United States; ^2^Anesthesiology and Surgical Oncology Research Group, Houston, TX, United States; ^3^Department of Biostatistics, The University of Texas MD Anderson Cancer Center, Houston, TX, United States

**Keywords:** regional anesthesia (RA), race, ethnicity, postoperative pain, opioids

## Abstract

**Background:**

Where applicable, regional anesthesia has been shown to be superior to opioid or non-opioid analgesic modalities alone. However, some studies have shown ethnic-based disparities in the use of regional anesthesia in patients undergoing surgical procedures. In this study of patients who had undergone major oncologic surgery, our main objective was to compare the use of regional anesthesia between patients of different ethnicities.

**Methods:**

A retrospective review of adults who had undergone major open abdominal surgical procedures between 2016 and 2021 was performed. Logistic regression models were used to assess the association between baseline patient characteristics and the use of regional anesthesia.

**Results:**

A total of 4,791 patients were included in the analysis. The median age was 60.5 years [interquartile range, 49, 69], the majority were female (65%), and of American Society of Anesthesiologists Physical Status Class (ASA) 3 (94.7%). Regional anesthesia was used in 2,652 patients (55.4%) and was not associated with race or ethnicity (*p* = 0.287). Compared to White patients, the odds of regional anesthesia use in other racial/ethnic groups were: Asian {odds ratio (OR) 0.851 [95% confidence interval (CI), 0.660–1.097]; *p* = 0.2125}, Black/African American [OR 0.807 (95% CI, 0.651–1.001); *p* = 0.0508], Hispanic/Latino [OR 0.957 (95% CI, 0.824–1.154); *p* = 0.7676], Other race [OR 0.957 (95% CI, 0.627–1.461); *p* = 0.8376]. In the multivariable analysis, age [OR 0.995 (95% CI, 0.991–1.000); *p* = 0.0309] and female gender [OR 1.231 (95% CI, 1.090–1.390); *p* = 0.0008] were associated with the use of regional anesthesia.

**Conclusion:**

In this single-institution retrospective study of adults who had undergone major open abdominal surgery, the use of regional anesthesia was not associated with race or ethnicity. In the multivariable analysis, age and female gender were associated with the use of regional anesthesia.

## Introduction

Racial and ethnic-based disparities in healthcare delivery have been long studied. These disparities are not only associated with higher morbidity and mortality among ethnic minorities from diseases such as diabetes, cardiovascular disease, and cancer, but have also been associated with a lesser likelihood of receiving optimal pain management (1–3).

The inclusion of regional anesthesia in perioperative pain control regimens has been shown to be superior to opioid or non-opioid analgesic modalities alone (4–6). However, some studies have shown ethnic-based disparities in the use of regional anesthesia (1, 7–11). For example, in a retrospective study of 639 patients in an enhanced recovery program, the use of epidural anesthesia or transversus abdominis plane (TAP) blocks was 13% lower in non-White patients than in White patients (1). In another retrospective cohort study of 5,810 adults who had undergone inguinal hernia repair, patients who identified as Black and those of other ethnic minority groups were up to 68% less likely to receive epidural anesthesia compared with their White counterparts (8). A similar observation was made in 81, 345 patients who had undergone mastectomy, where compared to White patients, the odds of receipt of regional anesthesia was up to 21% lower in non-White patients (9). Potential reasons for these disparities have included implicit bias (1, 8, 11), language barriers (10, 11), and cultural preferences (11).

On the other hand, other studies including some with very large cohorts, have not shown an association between race or ethnicity and the receipt of regional anesthesia. For example, in a retrospective propensity matched cohort study of patients in the American College of Surgeons-National Surgical Quality Improvement Program (ACS NSQIP) database, patient race or ethnicity was not associated with the type of anesthesia received for total joint arthroplasty (12). In another a single-center study of 25,664 children undergoing surgery at a tertiary children's hospital, race and ethnicity were not associated with the odds of receiving regional anesthesia (13). These differences in findings suggest ethnic-based disparities in the use of regional anesthesia may vary from institution to institution.

To effectively identify and address any such disparities, studies in different patient populations and at local and institutional levels are required. To the best of our knowledge, racial or ethnic-based differences in the use of regional anesthesia in patients undergoing major abdominal surgery for cancer has not been evaluated. To that end, we conducted a retrospective study of adult patients who had undergone major open abdominal surgery, with the primary objective of comparing the use of regional anesthesia (epidural or truncal blocks) between non-Hispanic White patients and patients of different races and ethnicities. Based on the results of previous studies (1, 8, 9), our hypothesis was that non-Hispanic White patients were more likely to receive regional anesthesia than patients of other racial or ethnic groups. The secondary objectives included racial or ethnic-based comparisons of intraoperative and immediate postoperative opioid administration, and early postoperative pain intensity scores.

## Materials and methods

This study was approved by the Institutional Review Board (IRB) of the University of Texas MD Anderson Cancer Center on September 27, 2021 (IRB # 2021-0738).

### Patient selection

**Figure 1:**
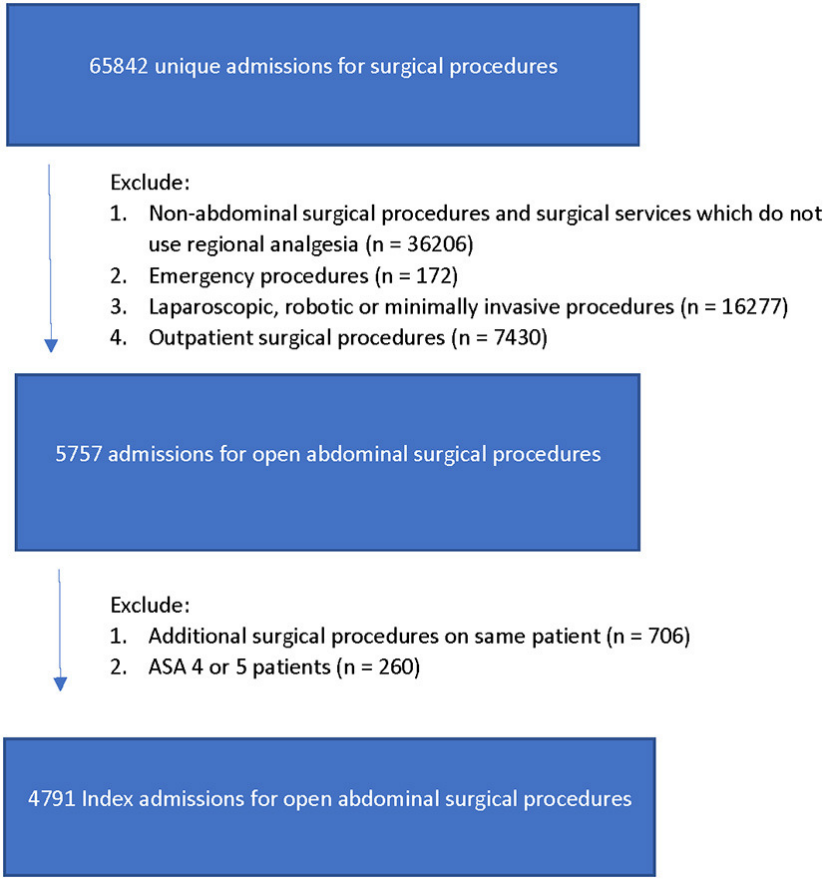
Patient selection process.

The institutional data warehouse was used to identify patient admissions for surgical procedures between March 1, 2016, and August 1, 2021. The patient selection process was designed to include only those patients who would have been offered a regional anesthetic preoperatively. Thus, non-abdominal procedures and those performed by surgical services who do not use regional anesthesia were excluded. Additionally, due to the lesser likelihood of use of epidural anesthesia or truncal blocks, patients of American Society of Anesthesiologists Physical Status (ASA PS) 4 and above, and those undergoing emergency and outpatient procedures were excluded. Furthermore, due to the higher likelihood of surgeon-performed local anesthetic block, laparoscopic and robotic assisted procedures were excluded. To avoid over-representation of individual patients, only data from their index admission for open abdominal surgery was evaluated. The patient selection process is illustrated in Figure 1.

### Clinical variables of interest

Perioperative variables were extracted from subsections of the institutional data warehouse including the Anesthesia, Pharmacy, Orders, Order Reconciliation, and Oncology Universes.

Patient demographics, a history of anxiety, depression, chronic pain, opioid use within the 3 months prior to surgery (preoperative opioid use), as well as any history of smoking, alcohol or drug abuse were recorded. Baseline coagulation parameters including platelet count, prothrombin time (PT), international normalized ratio (INR), and activated partial thromboplastin time (aPTT) were also recorded.

The use of epidural anesthesia or truncal blocks which were performed as a part of the initial anesthetic were recorded as “Regional Anesthesia” (Yes/No). The use of rescue blocks, and regional anesthetics which were performed postoperatively was not evaluated for this study. Intraoperative opioid administration, Post Anesthesia Care Unit (PACU) opioid consumption in morphine daily dose equivalents (MEDD), PACU pain intensity using verbal numeric rating scores (0 = no pain, 10 = worst pain ever), PACU length of stay (hours), and verbal numeric rating pain scores on postoperative day one were also recorded.

### Anesthetic and postoperative pain management

At our institution, the decision to use regional anesthesia or not is largely determined by our surgeons' established preferences. The type of regional anesthetic is also largely determined by surgeons' preferences. For the most part, regional anesthesia for open abdominal procedures involves either a thoracic epidural catheter or bilateral transversus abdominis plane (TAP) and quadratus lumborum blocks. Occasionally, paravertebral blocks and erector spinae plane blocks are used. All regional anesthetics which are performed for postoperative management are performed prior to the surgical incision. Other aspects of anesthetic management including intraoperative opioid administration and the use of multimodal analgesic techniques are not standardized. In particular, multimodal analgesic techniques are used to varying degrees by different practitioners.

In the PACU, a standardized order-set with preset dosages and limits for opioid and non-opioid analgesic medications is used. Additional doses are ordered for inadequate pain control. After discharge from the PACU, pain control in patients who did not receive regional anesthesia is managed by the surgical services according to service-based customized order-sets. Patients who received regional anesthesia continue to be managed by the acute pain service until regional anesthesia catheters have been discontinued, or adequate pain control has been established with the use of opioid and non-opioid analgesics. During this period, pain assessment is initially performed every hour for the first 12 h, then every 4 h thereafter. In addition, pain assessments are performed 1 h after any change in medication administration.

### Statistical analysis

Patients' demographics, treatment, and clinical outcomes were summarized through descriptive statistics. The Wilcoxon rank sum test or Kruskal-Wallis test was used to compare location parameters of continuous distributions between or among patient groups. The Chi-square test was used to evaluate the association between two categorical variables. A multivariable logistic regression model was fitted to estimate the effects of important covariates on regional anesthesia use and highest or average PACU pain score using 5 as the cutoff point. Statistical software SAS 9.4 (SAS, Cary, NC) and Splus 8.2 (TIBCO Software Inc., Palo Alto, CA) were used for all the analyses.

## Results

A total of 4,791 patients were included in the analysis. The median age [Interquartile Range (IQR)] was 60.5 years [IQR, 49, 69], the majority were female (65%), and of ASA class 3 (94.7%).

Information about race and ethnicity was missing for 34 patients. Among those patients with information, 257 (5.4%) were Asian, 373 (7.8%) were Black or African American, 652 (13.7%) were Hispanic or Latino, 3,391 (71.3%) were non-Hispanic White (White), and due the small numbers in their individual groups, 89 (1.9%) were classified together as Other race. Of the patients who were classified together as Other race, 66 (1.4%) self-identified as Other race, 17 (0.4%) as American Indian or Alaska Native, and 6 (0.1%) as Native Hawaiian or Other Pacific Islander.

Baseline characteristics of the study population are shown in Table 1. Compared to patients of other races or ethnicities, the group of White patients were older [median 61 years, interquartile range (IQR) 50, 69] and had the highest proportion of proportion of patients with a diagnosis of anxiety or depression (683/3,391 [20.1%]). Black or African American patients had the highest proportion of female patients (189/368 [51.4%]), and highest values for platelet counts (median 241, IQR [193, 305]), PT (median 13.7, IQR [13.1, 14.4]), and INR (median 1.04, IQR [1.0, 1.1]). The group of Asian patients had the highest median value for aPTT (median 31.1, IQR [29.2, 33.8]), and the lowest proportion of patients with a BMI > 25 (108/255 [42.4%]). Preoperative opioid use was highest within the group of patients categorized as “Other race”.

**Table 1:** Demographic and baseline characteristics of 4,791 adults undergoing open abdominal surgery.

**Baseline characteristics**	**All** **(*n* = 4,791)**	**Asian** **(*n* = 257)**	**Black or African American** **(*n* = 373)**	**Hispanic or Latino** **(*n* = 652)**	**Other** **(*n* = 89)**	**White** **(*n* = 3,391)**	***p*-value**
Age, years	60.0 [49, 69]	55 [45, 66]	59 [49, 67]	55 [44, 65]	55 [44, 66]	61 [50, 69]	<0.001
Gender, *n* (%)							0.027
Female	2,187 (45.7)	98 (38.1)	189 (51.4)	294 (45.1)	39 (43.8)	1,554 (45.8)	
Male	2,604 (54.3)	159 (61.9)	179 (48.6)	358 (54.9)	50 (56.2)	1,837 (54.2)	
Body mass index	27.6 [24.2, 31.7]	24.0 [21.9, 26.9]	29.4 [25.4, 33.5]	28.5 [24.8, 32.8]	27.3 [24.5, 30.8]	27.6 [24.2, 31.6]	<0.001
ASA, *n* (%)							0.144
1/II	256 (5.3)	18 (7.0)	17 (4.6)	45 (6.9)	7 (7.9)	169 (5)	
III/IV	4,535 (94.7)	239 (93.0)	351 (95.4)	607 (93.1)	82 (92.1)	169 (5)	
Anxiety/depression, *n* (%)							<0.01
Yes	905 (18.9)	17 (6.6)	58 (15.8)	129 (19.8)	14 (15.7)	683 (20.1)	
No	3,886 (81.1)	240 (93.4)	310 (84.2)	523 (80.2)	75 (84.3)	2,708 (79.9)	
Chronic pain, *n* (%)							0.765
Yes	20 (0.4)	1 (0.4)	2 (0.5)	1 (0.2)	0 (0)	16 (0.5)	
No	4,771 (99.6)	256 (99.6)	366 (99.5)	651 (99.8)	89 (100)	3,375 (99.5)	
Preop opioid use, *n* (%)							0.038
Yes	1,231 (25.7)	71 (27.6)	105 (28.5)	176 (27)	33 (37.1)	840 (24.8)	
No	3,560 (74.3)	186 (72.4)	263 (71.5)	476 (73)	56 (62.9)	2,551 (75.2)	
Smoking history, *n* (%)							0.301
Yes	12 (0.3)	0 (0)	2 (0.5)	1 (0.2)	1 (1.1)	8 (0.2)	
No	4,779 (99.7)	257 (100)	366 (99.5)	651 (99.8)	88 (98.9)	3,383 (99.8)	
Alcohol abuse, *n* (%)							0.717
Yes	76 (1.6)	3 (1.2)	5 (1.4)	7 (1.1)	2 (2.2)	58 (1.7)	
No	4,715 (98.4)	254 (98.8)	363 (98.6)	645 (98.9)	87 (97.8)	3,333 (98.3)	
Preop labs,							
Platelet count, K/uL	221 [179, 277]	214 [170, 267]	242 [193, 305]	227 [184, 288]	216 [179, 279]	219 [176, 272]	<0.001
PT, secs	13.4 [12.8, 14]	13.2 [12.7, 13.7]	13.7 [13.1, 14.4]	13.5 [12.9, 14.1]	13.5 [12.9, 14.1]	13.4 [12.8, 14]	<0.001
INR	1.02 [0.96, 1.08]	1.00 [0.95, 1.05]	1.04 [1.00, 1.11]	1.02 [0.97, 1.09]	1.02 [0.96, 1.08]	1.01 [0.96, 1.07]	<0.001
aPTT, secs	29.9 [27.7, 32.7]	31.1 [29.2, 33.8]	30.4 [27.9, 33.7]	30.3 [28.0, 33.0]	29.4 [27.1, 33.2]	29.8 [27.5, 32.4]	<0.001

Data expressed as median [interquartile range] unless otherwise indicated. BMI, Body Mass Index; ASA, American Society of Anesthesiologists Physical Status Score; Preop, Preoperative; PT, Prothrombin Time; INR, International Normalized Ratio; aPTT, activated Partial Thromboplastin Time.

### Use of regional anesthesia

Regional anesthesia was used in 2,652/4,791 patients (55.4%) and included epidural catheters (1,221/4,791, 25.5%), TAP/quadratus lumborum blocks (1,429/4,791, 29.8%), paravertebral blocks (1/4,791, 0.02%), and erector spinae plane blocks (1/4791, 0.02%). A larger proportion of females than males received regional anesthesia (58.4 vs. 52.8% males, *p* = 0.0001).

Patients who received regional anesthesia were also younger than those who did not receive regional anesthesia (median 59.5 years, IQR [48, 68], vs. 61 years, IQR [49, 69], *p* = 0.0029).

The use of regional anesthesia was not associated with statistically significant differences based on race or ethnicity (*p* = 0.287). The proportions of patients who received regional anesthesia within each racial or ethnic group were; Asian (52.1%), Black or African American (50.8%), Hispanic or Latino (55.5%), Other race (55.1%), and White (56.1%). The univariate analysis (Table 2) showed that age {odds ratio (OR) 0.995 [95% confidence interval (CI), 0.991–1.000]; *p* = 0.0296}, female gender [OR 1.259 (95% CI, 1.122–1.412); *p* < 0.0001], ASA class (56.3% of ASA 3 or higher vs. 38.3% of ASA 2; *p* < 0.001), and a history of anxiety or depression [OR 1.215 (95% CI, 1.049–1.407); *p* = 0.0093] were associated with the use of regional anesthesia. Higher values of PT [OR 0.838 (95% CI, 0.794–0.885); *p* < 0.0001], and INR [OR 0.335 (95% CI, 0.203–0.553); *p* < 0.0001] were associated with decreased odds for the receipt of regional anesthesia. The multivariate analysis indicated that only age [OR 0.995 (95% CI, 0.991–1.000); *p* = 0.0309] and female gender [OR 1.231 (95% CI, 1.090–1.390); *p* = 0.0008] were independent predictors of the use of regional anesthesia (Table 2).

**Table 2:** Association between baseline patient characteristics and the use of regional anesthesia.

	**Univariable analysis**			**Multivariable analysis**		
**Effect**	**OR estimate**	**95% CI for OR**	***p*-value**	**OR estimate**	**95% CI for OR**	***p*-value**
Asian vs. White	0.851	0.660, 1.097	0.2125	0.825	0.633, 1.074	0.1528
Black/AA vs. White	0.807	0.651, 1.001	0.0508	0.842	0.672, 1.055	0.1348
Hispanic/Latino vs. White	0.957	0.824, 1.154	0.7676	0.958	0.804, 1.140	0.6274
Other race vs. White	0.957	0.627, 1.461	0.8376	0.988	0.640, 1.524	0.9548
Age	0.995	0.991, 1.000	0.0296	0.995	0.991, 1.000	0.0309
Female vs. Male	1.259	1.122, 1.412	<0.0001	1.231	1.090, 1.390	0.0008
BMI	1.001	0.991, 1.010	0.8935	1.001	0.991, 1.011	0.8073
Platelet count	1.000	0.999, 1.001	0.7789	1.000	0.999, 1.000	0.4664
aPTT	0.995	0.981, 1.009	0.4716			
PT	0.838	0.794, 0.885	<0.0001			
INR	0.335	0.203, 0.553	<0.0001			
Anxiety or depression (Yes vs. No)	1.215	1.049, 1.407	0.0093	1.096	0.940, 1.278	0.2441
Chronic pain (Yes vs. No)	0.804	0.334, 1.936	0.6274			
Preoperative opioids (Yes vs. No)	1.071	0.940, 1.221	0.3006			
Alcohol abuse (Yes vs. No)	1.110	0.702, 1.757	0.6555	1.170	0.729, 1.877	0.5159
Smoking (Yes vs. No)	1.128	0.358, 3.560	0.8366			

AA, African American; BMI, Body Mass Index; aPTT, activated Partial Thromboplastin Time; PT, Prothrombin Time; INR, International Normalized Ratio.

### Opioid administration in the operating room and post anesthesia care unit

Intraoperative opioid administration was not associated with patient race or ethnicity (Table 3). However, opioid administration in the PACU was associated with race/ethnicity (*p* = 0.038) with the highest administration observed in patients who were categorized as Other race.

**Table 3:** Opioid administration and average pain scores of 4,791 adults undergoing open abdominal surgery for cancer.

	**All** **(*n* = 4,791)**	**Asian** **(*n* = 257)**	**Black or African American** **(*n* = 373)**	**Hispanic or Latino** **(*n* = 652)**	**Other** **(*n* = 89)**	**White** **(*n* = 3,391)**	***p*-value**
Intraoperative opioids, MEDD	35.6 (±27.8)	32.7 (±22.1)	37.3 (±26.7)	35.5 (±28.3)	36.9 (±24.3)	35.6 (±28.3)	0.151
PACU opioids, MEDD	9.3 (±9.0)	8.1 (±8.5)	9.8 (±7.7)	8.9 (±8.6)	10.2 (±9.1)	9.3 (±9.3)	0.038
Highest PACU pain score	5.9 (±2.9)	5.2 (±3.2)	6.5 (±2.9)	5.9 (±2.9)	6.2 (±2.9)	5.9 (±2.9)	<0.001
Average PACU pain score	2.7 (±1.8)	2.3 (±1.7)	3.0 (±1.9)	2.7 (±1.8)	2.7 (±1.9)	2.7 (±1.8)	<0.001
Highest POD # 1 pain score	5.7 (±2.5)	5.2 (±2.3)	6.1 (±2.5)	5.9 (±2.5)	6.0 (±2.6)	5.7 (±2.5)	<0.001
Average POD #1 pain score	2.8 (±1.6)	2.5 (±1.5)	3.0 (±1.7)	2.9 (±1.7)	2.8 (±1.5)	2.8 (±1.6)	0.002

MEDD, Morphine Equivalent Daily Dose; PACU, Post Anesthesia Care Unit; POD, Postoperative Day.

### Postoperative pain scores

Pain intensity in the PACU was associated with race and ethnicity (*p* < 0.001). The highest and average PACU pain scores were significantly lower in Asian patients, and highest in Black or African American Patients (Table 3). Pain intensity on postoperative day one was also significantly associated with race/ethnicity. Similar to pain intensity in the PACU, the highest and average pain scores on postoperative day one were significantly lower in the group of Asian patients, and highest in the group of Black or African American patients (Table 3).

Regarding highest PACU pain scores ≥ 5, patients who were 60 years of age or older (1,646/2,468 [66.7%]; *p* < 0.001), male patients (1,777/2,599 [68.4%]; *p* < 0.001), Asian patients (154/257 [59.9%]; *p* = 0.0001), those without a history of anxiety or depression (2,713/3,881 [69.9%]; *p* < 0.001), and those who did not use opioids prior to surgery (2,464/3,559 [69.2%]; *p* < 0.001) had significantly lower proportions of patients with a highest PACU pain score of 5 or higher (Table 4). In the multivariable analysis (Table 5), the association between race/ethnicity and a highest PACU pain score ≥ 5 was significant (*p* < 0.001). In this regard, compared to White patients, Asian patients had a significantly lower likelihood of having a highest PACU pain score of 5 or greater [OR 0.581 (95% CI, 0.443–0.762); *p* < 0.001], and Black or African American patients had greater than a 30% likelihood of having a score of 5 or greater [OR 1.384 (95% CI, 1.066–1.797); *p* = 0.015]. Furthermore, patients who used opioids preoperatively [OR 1.372 (95% CI, 1.178–1.598); *p* < 0.001], and those with a history of anxiety or depression [OR 1.229 (95% CI, 1.034–1.461); *p* = 0.019] had greater odds of having a highest PACU pain score of 5 or greater. Additionally, the likelihood of having a highest PACU pain score ≥ 5 lessened with increasing patient age [OR 0.917 (95% CI, 0.895–0.939); *p* < 0.001].

**Table 4:** Univariable analysis of the association between highest and average PACU pain scores and the perioperative characteristics of 4,791 adults undergoing open abdominal surgery.

**Variable, *n* (%)**	**Highest pain in PACU <5**	**Highest pain in PACU ≥5**	***p*-value**	**Average pain in PACU <5**	**Average pain in PACU ≥5**	***p*-value**
Age			<0.0001			<0.0001
<60	562 (24.2)	1,756 (75.8)		1,966 (84.8)	352 (15.2)	
≥60	822 (33.3)	1,646 (66.7)		2,263 (91.7)	205 (8.3)	
Gender			<0.0001			0.0268
Female	562 (25.7)	1,625 (74.3)		1,908 (87.2)	279 (12.8)	
Male	822 (31.6)	1,777 (68.4)		2,321 (89.3)	278 (10.7)	
Race/ethnicity			0.0001			0.0004
Asian	103 (40.1)	154 (59.9)		239 (93)	18 (7)	
Black/African American	81 (22.1)	285 (77.9)		300 (82)	66 (18)	
Hispanic or Latino	181 (27.8)	470 (72.2)		577 (88.6)	74 (11.4)	
Other	23 (25.8)	66 (74.2)		78 (87.6)	11 (12.4)	
White	987 (29.1)	2,402 (70.9)		3,004 (88.6)	385 (11.4)	
ASA			0.6738			0.5757
I/II	77 (30.1)	179 (69.9)		229 (89.5)	27 (10.5)	
III	1,307 (28.9)	3,223 (71.1)		4,000 (88.3)	530 (11.7)	
BMI			0.9055			0.0690
>25	953 (28.9)	2,348 (71.1)		1,316 (89.7)	151 (10.3)	
≤ 25	426 (29)	1,041 (71)		2,901 (87.9)	400 (12.1)	
Anxiety or depression			0.0002			<0.0001
Yes	216 (23.9)	689 (76.1)		751 (83)	154 (17)	
No	1,168 (30.1)	2,713 (69.9)		3,478 (89.6)	403 (10.4)	
Chronic pain			0.1689			0.0619
Yes	3 (15)	17 (85)		15 (75)	5 (25)	
No	1,381 (29)	3,385 (71)		4,214 (88.4)	552 (11.6)	
Preoperative opioids			<0.0001			<0.0001
Yes	289 (23.6)	938 (76.4)		987 (80.4)	240 (19.6)	
No	1,095 (30.8)	2,464 (69.2)		3,242 (91.1)	317 (8.9)	
Alcohol abuse			0.4408			0.1341
Yes	25 (32.9)	51 (67.1)		63 (82.9)	13 (17.1)	
No	1,359(28.9)	3,351 (71.1)		4,166 (88.5)	544 (11.5)	
Smoker			0.7644			0.1484
Yes	3 (25)	9 (75)		9 (75)	3 (25)	
No	1,381 (28.9)	3,393 (71.1)		4,220 (88.4)	554 (11.6)	
Regional anesthesia			0.5377			0.2959
Yes	757 (28.6)	1,894 (71.4)		2,354 (88.8)	297 (11.2)	
No	627 (29.4)	1,508 (70.6)		1,875 (87.8)	260 (12.2)	
Intraoperative opioids, MEDD (mean ± SD)	34 ± 28	36 ± 27	0.0007	34 ± 25	45 ± 41	<0.0001
PACU opioids, MEDD (mean ± SD)	2 ± 5	11 ± 9	<0.0001	8 ± 7	18 ± 12	<0.0001
PACU duration, hrs (mean ± SD)	3 ± 2	4 ± 2	<0.0001	4 ± 2	4 ± 2	0.1813

ASA, American Society of Anesthesiologists Physical Status Class; BMI, Body Mass Index; MEDD, Morphine Equivalent Daily Dose; PACU, Post Anesthesia Care Unit.

**Table 5:** Multivariable analysis of the association between highest and average PACU pain scores and the perioperative characteristics of 4,791 adults undergoing open abdominal surgery.

**Multivariable analysis for PACU pain scores**								
	**Highest PACU pain** ≥**5**				**Average PACU pain** ≥**5**			
**Effect**	**OR estimate**	**95% CI**		***p*-value**	**OR Estimate**	**95% CI**		***p*-value**
Age	0.917	0.895	0.939	<0.0001	0.894	0.865	0.924	<0.0001
BMI	0.998	0.987	1.009	0.7621	1.022	1.008	1.037	0.0027
Female vs. male	1.259	1.105	1.435	0.0006	1.098	0.911	1.322	0.3261
Asian vs. White	0.581	0.443	0.762	<0.0001	0.594	0.359	0.985	0.0436
Black/African American vs. White	1.384	1.066	1.797	0.0148	1.617	1.203	2.175	0.0015
Hispanic/Latino vs. White	0.977	0.807	1.183	0.8102	0.849	0.645	1.117	0.2417
Other vs. White	1.067	0.656	1.735	0.7934	0.921	0.478	1.776	0.8064
ASA III vs. I/II	1.146	0.863	1.524	0.3463	1.211	0.787	1.862	0.3836
Preoperative opioids, Yes vs. No	1.372	1.178	1.598	<0.0001	2.416	2.003	2.914	<0.0001
Regional anesthesia, No vs. Yes	1.010	0.888	1.149	0.8786	1.156	0.962	1.389	0.1226
Anxiety/depression Yes vs. No	1.229	1.034	1.461	0.0193	1.569	1.268	1.940	<0.0001

ASA, American Society of Anesthesiologists Physical Status Class; BMI, Body Mass Index.

The proportion of patients with an average PACU pain score of 5 or higher was significantly lower among patients who were 60 years of age or older (205/2,468 [8.3%]; *p* < 0.001), male patients (278/2,599 [10.7%]; *p* < 0.027), Asian patients (18/257 [7%]; *p* = 0.0004), those without a history of anxiety or depression (403/3,881 [10.4%]; *p* < 0.001), and those who did not use opioids prior to surgery (317/3,559 [8.9%]; *p* < 0.001). The multivariable model (Table 5) demonstrated a significant association between race/ethnicity and an average PACU pain score of 5 or higher (*p* = 0.0015). Compared to White patients, Asian patients had a lesser likelihood of having an average pain score of 5 or higher [OR 0.594 (95% CI, 0.359–0.985); *p* = 0.044]. On the other hand, Black or African American patients had a >60% likelihood of having an average PACU pain score of 5 or higher [OR 1.617 (95% CI, 1.203–2.175); *p* = 0.002]. Patient age [OR 0.894 (95% CI, 0.865–0.924); *p* < 0.001], BMI [OR 1.002 (95% CI, 1.008–1.037); *p* = 0.003], preoperative opioid use [OR 2.416 (95% CI, 2.003–2.914); *p* < 0.001], and a history of anxiety and/or depression [OR 1.569 (95% CI, 1.268–1.940); *p* < 0.001] were also independently associated with an average PACU pain score of 5 or higher.

## Discussion

In this single-center retrospective study, there were no statistically significant racial or ethnic-based differences in the use of regional anesthesia or in intraoperative opioid administration. However, significant racial and ethnic-based differences were observed in terms of postoperative pain intensity and in the administration of opioids in the PACU. In this regard, the severity of postoperative pain was lowest in the group of Asian patients and highest in Black or African American patients. Postoperative opioid administration was highest in patients who were grouped together as “Other Race”.

Similar to our findings, the absence of an association between the use of regional anesthesia and patient race or ethnicity has been reported in other patient populations (12, 13). For example, in a retrospective cohort study by Elsharydah et al. (12), the proportion of African American patients who underwent total hip and knee arthroplasty with regional anesthesia was 2.3% less than in White patients. However, this observed difference was not detectable after propensity score matching. Similarly, in a large single-center study of pediatric patients, the proportion of minority patients who received regional anesthesia for their procedures was 1.4% less than their White counterparts. However, there was no statistically significant difference after multivariable and sensitivity analyses.

One of the major challenges in addressing racial or ethnic-based disparities in healthcare delivery is the difficulty in determining the reasons for its existence or absence. Regarding our study, the decision to use regional anesthesia or not was largely based on individual surgeons' established preferences. On any given day, modifications or changes to this established preference was discussed between the surgeon and the anesthesiologist. We speculate that this added level of discussion may have aided in ameliorating any potential racial or ethnic-based biases in offering regional anesthesia to patients.

In this study, younger age and female gender were independently associated with higher odds of receiving regional anesthesia. The reasons for these significant associations are not discernible from our data. With regard to age, the difference in age between the study groups, although statistically significant, may not be clinically significant. Thus, it is difficult to speculate about possible reasons for this statistical significance. With regard to female patients having higher odds of receiving regional anesthesia, a survey investigating patient perceptions of regional anesthesia revealed that more patients, especially females, would accept regional anesthesia if reassured appropriately (14). Furthermore, patients were more likely to accept regional anesthesia if they had chosen it in the past. Based on the findings of this survey, it may be possible that prior experience with labor epidurals contributed to a higher rate of acceptance of regional anesthesia among females patients in our study population.

In our study population, postoperative pain intensity was statistically different based on race and ethnicity. In this regard, Asian patients had the lowest and Black or African American had the highest pain scores in the PACU and on postoperative day one. The results of studies evaluating postoperative and experimental pain in the Asian population have been mixed (15–18). These mixed results may be due to the complex interaction of cultural, social, biologic and genetic factors. The diverse nature of the population on the Asian continent may also contribute to the mixed findings.

On the other hand, several studies have demonstrated that Black patients have a lower threshold to painful stimuli and report more postoperative pain than White patients (16, 17, 19). Some have reported on this disparity even when regional anesthesia has been used (20). This higher burden of pain has been attributed to physiological, social, cultural and provider-level reasons (21).

In our study, higher BMI was independently associated with greater odds of an average PACU score of 5 or higher. Furthermore, BMI was significantly associated with race and ethnicity, with the group of Black or African American patients having the highest median BMI among all racial or ethnic groups. The association between BMI and postoperative pain has been reported by other studies as well (22, 23). Postulated mechanisms for the decreased effectiveness of regional anesthetic techniques in obese patients include an increased rate of failure to accurately identify anatomical landmarks (23, 24), and altered pharmacokinetics of local anesthetics in adipose tissue (22). Other authors have suggested that compared to ultrasound guided transversus abdominis plane block, ultrasound guided erector spinae block may be more feasible and effective in providing intra and postoperative analgesia in patients with high BMI (25).

Other factors which were independently associated with a higher intensity of postoperative pain included younger patient age, female gender, preoperative opioid use, and a history of anxiety or depression. In a recent systematic review and meta-analysis representing 53,362 patients, Yang et al. identified similar factors to be predictive of poor acute postoperative pain control (26). In the current study, despite having significantly higher odds of receiving regional anesthesia, younger patients and female patients had significantly higher pain intensity. This finding suggests that other measures may have been necessary to attain adequate pain control in this group of patients. For example, in women undergoing breast cancer surgery, preoperative interventions such as music therapy and aromatherapy have been shown to be effective in reducing preoperative anxiety, whilst music therapy and acupuncture were shown to be effective in minimizing postoperative pain (27). With regard to preoperative anxiety, preoperative complimentary therapies such as music therapy, aromatherapy and guided imagery have been shown to reduce preoperative anxiety, albeit to varying degrees (27, 28).

With regard to the association between preoperative opioid use and higher postoperative pain intensity, chronic opioid use has been associated with tolerance to opioids and opioid induced hyperalgesia (OIH), both of which could result in higher postoperative pain intensity and increased opioid requirements (29, 30). In our study, the group of patients who were classified as “Other” race had a significantly higher proportion of patients who used opioids preoperatively. Accordingly, postoperative opioid requirements were significantly higher in this sub-group of patients. The molecular mechanisms of tolerance and OIH may be due to neuroplastic changes in the peripheral and central nervous systems that result in sensitization of pronociceptive pathways, and the N-methyl-D-aspartate (NMDA) receptor system has been shown to play a significant role (31). The inclusion of NMDA receptor modulators such as methadone and ketamine in pain control regimens has been shown to reduce opioid usage and improve pain control in patients who may be tolerant to opioids and in those who are susceptible to OIH (32, 33).

This study has several limitations. Firstly, the retrospective nature of this study meant details of the decision to use or not to use regional anesthesia could not be determined with certainty. Second, several missing values for platelet counts, PT, INR, and aPTT meant they could not be included in the multivariable analysis to determine their effect on the use of regional anesthesia. Lastly, the lack of available studies on ethnic disparities in the use of regional anesthesia during major abdominal surgery meant we could not perform an *a priori* sample-size analysis.

In conclusion, in this single-center retrospective study of adults who had undergone major abdominal surgery for cancer, the use of regional anesthesia was not associated with patient race or ethnicity. However, postoperative pain intensity and PACU opioid consumption were associated with race/ethnicity with the group of Asian patients having significantly lower pain scores, and the group of patients classified together as “Other race” having the highest PACU opioid consumption.

## Data availability statement

The raw data supporting the conclusions of this article will be made available by the authors, without undue reservation.

## Ethics statement

The studies involving human participants were reviewed and approved by Institutional Review Board (IRB) of the University of Texas MD Anderson Cancer Center (IRB # 2021-0738). Written informed consent for participation was not required for this study in accordance with the national legislation and the institutional requirements.

## Author contributions

PO-A: conceptualization of study, data collection, and writing of manuscript. JC: conceptualization of study, critical review of data, and writing of manuscript. VP and UW: critical review of data and writing of manuscript. LF: statistical analysis. All authors contributed to the article and approved the submitted version.

## Conflict of interest

The authors declare that the research was conducted in the absence of any commercial or financial relationships that could be construed as a potential conflict of interest.

## Publisher's note

All claims expressed in this article are solely those of the authors and do not necessarily represent those of their affiliated organizations, or those of the publisher, the editors and the reviewers. Any product that may be evaluated in this article, or claim that may be made by its manufacturer, is not guaranteed or endorsed by the publisher.
